# Effect of Intermittent Theta Burst Stimulation on the Neural Processing of Emotional Stimuli in Healthy Volunteers

**DOI:** 10.3390/jcm10112449

**Published:** 2021-06-01

**Authors:** Virginie Moulier, Christian Gaudeau-Bosma, Fanny Thomas, Clémence Isaac, Maxence Thomas, Florence Durand, Palmyre Schenin-King Andrianisaina, Romain Valabregue, Charles Laidi, René Benadhira, Noomane Bouaziz, Dominique Januel

**Affiliations:** 1 Unité de Recherche Clinique, EPS Ville-Evrard, 93332 Neuilly-sur-Marne, France; fanny.thomas1@gmail.com (F.T.); clm.isaac@gmail.com (C.I.); maxence.thomas@gmail.com (M.T.); durand.florence@hotmail.com (F.D.); palmyresking@hotmail.fr (P.S.-K.A.); reneben4@hotmail.com (R.B.); bouaziznoomane@gmail.com (N.B.); domjanuel@gmail.com (D.J.); 2 Centre Hospitalier du Rouvray, University Department of Psychiatry, 76301 Sotteville-lès-Rouen, France; 3 Espace Territoriale d’Accompagnement Psychosociale, CH Les Murets, GHT94, 94120 Fontenay sous Bois, France; gaudeau@gmail.com; 4 Institut du Cerveau et de la Moelle Épinière—ICM, Sorbonne Universités, UPMC Univ Paris 06, Inserm U1127, CNRS UMR 7225, 75013 Paris, France; romain.valabregue@upmc.fr; 5 Pôle de Psychiatrie, Assistance Publique-Hôpitaux de Paris (AP-HP), Faculté de Médecine de Créteil, DMU IMPACT, Hôpitaux Universitaires Mondor, 94028 Créteil, France; charleslaidi@gmail.com; 6 Institut National de la Santé et de la Recherche Médicale (INSERM), U955, Institut Mondor de Recherche Biomédicale, Psychiatrie Translationnelle, 94028 Créteil, France; 7 UNIACT, Psychiatry Team, Neurospin Neuroimaging Platform, CEA Saclay, 91191 Gif-sur-Yvette, France; 8 Fondation Fondamental, 94028 Créteil, France

**Keywords:** iTBS, rTMS, emotion, brain, healthy subjects

## Abstract

Background: Intermittent theta burst stimulation (iTBS) is a form of repetitive transcranial magnetic stimulation that has shown to be effective in treatment-resistant depression. Through studying the effect of iTBS on healthy subjects, we wished to attain a greater understanding of its impact on the brain. Our objective was to assess whether 10 iTBS sessions altered the neural processing of emotional stimuli, mood and brain anatomy in healthy subjects. Methods: In this double-blind randomized sham-controlled study, 30 subjects received either active iTBS treatment (10 sessions, two sessions a day) or sham treatment over the left dorsolateral prefrontal cortex. Assessments of mood, structural magnetic resonance imaging (MRI) and functional MRI (fMRI) were performed before and after iTBS sessions. During the fMRI, three different categories of stimuli were presented: positive, negative and neutral photographs. Results: This study showed that, during the presentation of negative stimuli (compared with neutral stimuli), 10 sessions of iTBS increased activity in the left anterior insula. However, iTBS did not induce any change in mood, regional gray matter volume or cortical thickness. Conclusions: iTBS modifies healthy subjects’ brain activity in a key region that processes emotional stimuli. (AFSSAPS: ID-RCB 2010A01032-37).

## 1. Introduction

Repetitive transcranial magnetic stimulation (rTMS) is a noninvasive brain stimulation technique allowing various applications, such as disease diagnosis, investigation of cortical excitability changes, mapping of cortical function and therapeutics [1]. This technique uses electromagnetic pulses to induce a brief electrical current in the underlying cortical tissue. Rapid variation of the magnetic field and the induced electrical current generate an action potential which propagates along the neuronal synaptic chain. Repetitive application of this stimulation affects cortical activity (inhibition or excitation), generating modulation effects within the target region and its associated network [2]. Of the brain areas targeted in psychiatry, the dorsolateral prefrontal cortex (DLPFC) has received the most attention, as this area has long been at the center of pathophysiological models of depression [3].

In 2018, the Food and Drug Administration (FDA) approved a theta burst stimulation (TBS) protocol on the left DLPFC to alleviate depressive symptoms. TBS, a form of rTMS, is characterized by a burst of three 50-Hz pulses, which is repeated at intervals of 200 ms or 5 Hz, i.e., at the theta frequency [4]. Similar to high-frequency rTMS, intermittent TBS (iTBS) induces an increase in cortical excitability [5], but the duration of iTBS is shorter than in standard rTMS [6], which could improve its accessibility, acceptability and cost-effectiveness.

Even though early studies, which were based on small samples and not sham-controlled, reported significant effects of a single session of rTMS on mood in healthy individuals [7,8,9,10,11], a review highlighted that recent sham-controlled studies failed to find any changes in mood [12,13,14,15,16,17,18,19], leading the authors to conclude that a single session of rTMS has no impact on mood in healthy subjects [20]. Regarding the effect of multiple sessions, nine sessions of 25 Hz rTMS over the left DLPFC resulted in a significant reduction in Beck Depression Inventory (BDI) scores [21]. On the other hand, 10 rTMS sessions of 10 Hz over the left DLPFC [22] and two sessions of iTBS [23] did not show any effect on mood in healthy subjects.

Although subjectively experienced mood constitutes an important output of rTMS’s effect, it gives only limited insight into the neurocircuitry of emotion, as brain reactions to emotional stimuli are able to operate independently of verbal reports [13]. Mood and processing the emotional content of stimuli are distinct but related phenomena and can influence each other [24].

Although two studies showed no effect of high-frequency rTMS session(s) over the left DLPFC in the attentional processing of emotional information [25] or in the recognition of facial expressions of emotions [26], the administration of 5 Hz rTMS over the left DLPFC induced faster recognition of positive and high-arousing information during a memory task in 69 healthy participants [27].

Several studies also showed that administering rTMS could induce changes in brain activity during the presentation of emotional stimuli. One 10 Hz rTMS session over the left DLPFC resulted in diminished attentional towards angry faces in healthy women [28]. This effect was associated with activation in the left orbitofrontal cortex, the right DLPFC, the left anterior cingulate gyrus and the right superior parietal gyrus. In addition, one session of 10 HzrTMS over the left DLPFC increased activity in the left superior frontal gyrus and in the right inferior parietal lobule in women during the presentation of positive baby faces [16]. When negative baby faces were presented, rTMS induced decreased activity in the right insula. The regions whose activity was modified were cortical, more or less distant from the target of rTMS and known for their implication in the processing of emotions.

Until now, few studies have investigated the effect of TBS on emotion processing. First, Cao et al. assessed the effect of continuous TBS (cTBS) on brain activity during emotion processing in healthy subjects [29]. One session of cTBS over the right DLPFC decreased EEG alpha power during the presentation of happy faces. Since Coan and Allen pointed out that activity in the alpha range is inversely related to the underlying cortical processing [30], the authors suggested that cTBS increases brain activity for positive stimuli. More recently, several studies investigated the effects of one or two iTBS sessions on mood and/or emotion processing in healthy volunteers with parameters commonly used when treating mood disorders [23,31,32,33,34], which usually favor iTBS over the left DLPFC (Table 1).

These studies provided some evidence that iTBS does not alter mood but affects emotion processing in healthy individuals.

According to Harmer et al., antidepressant treatments may be underlaid by a rapid change in emotion processing that then leads to a slower improvement in mood [35]. The antidepressant effect of iTBS may result from emotion processing changes [33]. Assuming that the therapeutic mechanism of iTBS shares the same mechanism in healthy subjects, we suggest that repeated iTBS sessions over the left DLPFC over several days could modulate brain activity during emotion processing.

Consequently, in the current study, we wanted to assess whether 10 iTBS sessions applied over the left DLPFC altered the neural processing of emotional stimuli in healthy volunteers. By studying healthy subjects, we would be able to identify more precisely the brain correlates underlying the effect of iTBS, independently of depressive disorder, comorbidities and associated pharmacological treatments. For exploratory purposes, the (short- and long-term) effects of iTBS on mood and brain anatomy (regional gray matter density and cortical thickness) were also studied.

## 2. Materials and Methods

### 2.1. Participants

Thirty healthy right-handed volunteers between 18 and 60 years old were included in the study, which took place in the Department of Clinical Research at EPS Ville Evrard, Neuilly-sur-Marne, France. Participants were recruited through a mailing list about neuroscience research from March 2012 (https://www.risc.cnrs.fr/, accessed on 23 April 2021). The recruitment process lasted 26 months, or 29 months, including the follow-up period.

Subjects with current or previous neurological or psychiatric disorders, who were pregnant, who reported current physical disorders, who were on a course of pharmacological treatment, or who had a contraindication to magnetic resonance imaging (MRI), were excluded. Pregnancy blood tests were performed to confirm the absence of pregnancy. The Beck Depression Inventory (BDI) [36] and the Hamilton Depression Rating Scale (HDRS) [37] were administered, and subjects had to score below 8 to ensure the absence of depressive symptoms. Handedness was assessed using the Edinburgh Inventory [38]. Volunteers also had to be TMS-naive (i.e., never having received TMS sessions before) in order to respect the double-blind design. Subjects gave written informed consent to participate and received financial compensation for their participation. The local ethics committee approved the study (CPP Ile de France VIII, number 101078, ID-RCB 2010A01032-37).

### 2.2. Study Design

A 2-parallel-arm double-blind randomized trial was conducted. Volunteers were randomized to an active (*n* = 14) or sham (*n* = 16) rTMS arm with an allocation ratio of 1:1. Computer-generated randomization was implemented by an engineer using Microsoft Excel (2011) and was based on the Minimization Method with 2 controlled factors: gender and age [39]. MRI sessions and clinical assessments were performed before and after 10 active or sham rTMS sessions, which were administered in 1 week. Post-treatment assessments took place the following week: clinical assessment occurred 4 days after and MRI sessions 5 days after the last rTMS session. No rTMS was administered during MRI sessions. Regarding clinical assessment, long-term follow up took place 15 days and 3 months after the end of the stimulation. Volunteers, psychiatrists, psychologists and the researchers who led the MRI sessions were blind to the arm. Only the nurse who administered the rTMS sessions was aware of the allocated arm.

### 2.3. rTMS

**Figure 1:**
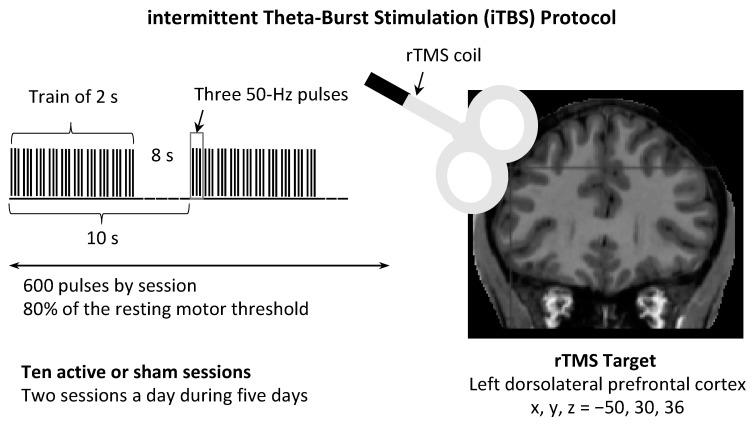
iTBS protocol applied over the left dorsolateral prefrontal cortex (DLPFC). Notes: The theta burst stimulation pattern consisted of three pulses of stimulation given at 50 Hz, repeated every 200 ms. In iTBS, a 2-second train of theta burst stimulation was repeated every 10 seconds for a total of 600 pulses. The coordinates (expressed in millimeters) of the rTMS target are defined in the MNI stereotactic space (Montreal Neurological Institute, Canada).

A Magstim Super Rapid stimulator (Magstim, Wales) was used with a 70 mm double air film coil. For the control group, a sham coil providing the same acoustic sensation, visual impact and shape as the active coil was used. This sham coil was equipped with a magnetic shield that attenuated the magnetic field to a biologically inactive level [40] but stimulated the skin and muscle overlying the scalp, giving the subjects the sensation of magnetic stimulation. The coil was located tangentially to the scalp over the left DLPFC using MRI-guided neuronavigation with Brainsight software (Rogue Research Inc., Canada). In order to optimize the coils’ placement and decrease inter-subject and inter-session variability, the left DLPFC was targeted using MNI coordinates (x, y, z = −50, 30, 36) that corresponded to the juncture of Brodmann areas (BA) 9 and 46 [41]. This brain area was labeled on a 3D rendering of each subject’s T1 MRI sequence using Statistical Parametric Mapping software (SPM12, Wellcome Department of Cognitive Neurology, London, UK). iTBS consisted of a burst of 3 50-Hz pulses, which was repeated at intervals of 200 ms [4]. A 2-s train of TBS was repeated every 10 s for a total of 600 pulses in 190 s (Figure 1). In order to define TMS intensity parameters, each subject’s individual motor threshold, considered to be an indicator of cortical excitability, was determined. The motor threshold can be estimated when the target muscle is at rest (rMT) or during active contraction of the target muscle (aMT). In the present study, 80% of the resting motor threshold (rMT) was applied instead of 80% of the active motor threshold [4], allowing higher stimulation power, while being well tolerated [42]. The use of rMT instead of aMT is also methodologically easier, since TMS interferes with the ability to maintain steady muscle contraction with a stable background EMG activity of 10–20% of maximal contraction [43]. The rMT was defined as the lowest TMS intensity required to induce a motor-evoked potential with an amplitude of at least 50 microvolts in at least 5 out of 10 trials [44]. In total, volunteers received 10 active or sham rTMS sessions: 2 sessions a day at 1 hour intervals were delivered every working day for 1 week.

### 2.4. Clinical Assessment

A clinical assessment of mood was performed before and after 10 rTMS sessions using the BDI, HDRS, Hospital Anxiety Depression Scale (HAD) [45] and the Bech–Rafaelsen Mania Scale (MAS) [46]. French versions of Norris’ Visual Analogue Scales (VAS) were used to measure drowsiness, daydreaming, energy, clarity of mind, clumsiness, vivacity, weakness, boredom, competency, sadness, peace of mind, dissatisfaction, sociability, restlessness, relaxation, quality of life and coping [47,48]. Another follow-up, using the HDRS, took place 15 days after the end of the stimulation, and the final follow up, using the BDI, took place 3 months after.

### 2.5. MRI Data Acquisition

Acquisitions were performed using a 3T Verio MRI scanner (Siemens, Erlangen, Germany). First, a 3D sequence was conducted for TMS neuronavigation, Voxel-Based Morphometry (VBM) and cortical thickness analyses, and localization of functional MRI (fMRI) activation (176 contiguous slices, slice thickness = 1.0 mm, TR = 2300 ms, TE = 2.98 ms, field of view = 256 × 256). Next, a functional run was acquired during an experimental task using a gradient echo-planar imaging (EPI) sequence (40 slices separated by a 1 mm inter-slice gap, slice thickness = 2 mm, TR = 2500 ms, TE = 25 ms, flip angle = 90°, field of view = 208 mm, number of volumes = 195, duration = 8 minutes 7.5 seconds). To allow for T1 equilibrium, each run started with 4 dummy scans, which were later discarded before analysis.

### 2.6. Experimental Task

The experimental task was programmed using Cogent 2000 software (Matlab). Three types of photographs were presented: (i) positive, being happy images; (ii) negative, being sad images, and (iii) neutral images. The photographs came from the International Affective Picture System (IAPS) [49] and from royalty-free photo libraries. They had previously been selected by an independent sample of 20 healthy participants. They rated 257 pictures in terms of valence (from 1 = highly negative to 9 = highly positive) and intensity (from 1 = very low to 9 = very high) with a 9-point scale. After having transformed the valence scores into z scores, the 40 images with the highest z scores were selected for the positive category, the 40 images with the lowest z scores for the negative category, and the 40 images with z scores closest to 0 for the neutral category. The pictures were projected onto a screen that the participants could see through a mirror. In order to check the alertness of the participants, they were requested to press a button whenever they saw a light signal inserted into 1 of the blocks of each category.

**Figure 2:**
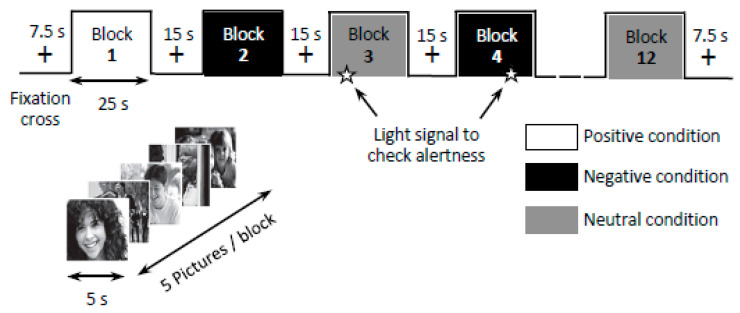
Experimental design of the fMRI session.

As illustrated in Figure 2, the functional run consisted of 12 blocks of 25 s, 4 for each of the 3 categories. Between each block, a 15-s fixation cross-block was presented. Within each block, 5 pictures of the same category were presented for 5 s each. During the run, in total, 20 pictures per category was presented. The run began and ended with a 7.5-s fixation cross-block. After the functional run, subjects rated each picture in terms of intensity from 1 (very low) to 9 (very high). Pictures presented during the first (before iTBS) and the second (after iTBS) MRI sessions were different for the same participant. To prevent activation of brain areas involved in memory processes and to maintain the same degree of novelty, different sets of photographs were shown in the first and second sessions. The order in which pictures were shown was counterbalanced between sessions and participants.

### 2.7. Voxel-Based Morphometry (VBM)

Three-dimensional structural images were pre-processed using SPM12. The images were segmented into different tissue classes with modulation and were then smoothed with an 8 mm full width at half-maximum Gaussian kernel. Next, a flexible factorial model was performed using tissue maps corresponding to gray matter, with time (before/after rTMS) as a within-subject factor and group (active/sham rTMS) as a between-group factor.

### 2.8. Cortical Thickness

Three-dimensional structural images were processed using FreeSurfer software (version 5.3.0; [50]; https://surfer.nmr.mgh.harvard.edu/, accessed on 23 April 2021). FreeSurfer automatically computed subject-specific measurements (such as cortical thickness) of the regions of interest (ROIs), which were labeled using the Desikan atlas [51]. These ROIs were located either in the region targeted by iTBS (the left middle frontal gyrus) or in regions that showed a change in their activity during fMRI.

### 2.9. Functional MRI Data Preprocessing and Analysis

Using SPM8, functional images were spatially realigned to compensate for subjects’ movement. The 3D structural image was coregistered using the mean functional image as a reference, and functional and 3D structural images were coregistered using the EPI template as a reference. Next, functional and 3D structural images were normalized using the transformation computed for the segmentation of structural images. Functional data was finally smoothed using an 8 mm full width at half-maximum Gaussian kernel. Analyses based on the general linear model were performed. In this approach, for each subject and for each volume element (voxel) of the brain, a general linear model was used to explain the level of the blood oxygenation level-dependent (BOLD) signal Y in terms of the linear combination of L explanatory variables (x_1_, x_2_,,...,x_L_), plus an error term [52]. A block model was specified with 3 explanatory variables (or regressors) related to the different categories of stimuli (positive, negative or neutral), 1 regressor associated with the light signal, and 6 motion parameters. Regressors were convolved with a canonical Hemodynamic Response Function. A high-pass filter (cut-off period: 256 seconds) was applied. First, individual analyses were performed in order to identify regions with a higher response to positive and/or negative stimuli compared with neutral stimuli. These first-level analyses produced statistical parametric maps for each subject. A whole-brain random-effects analysis (RFX), using a flexible factorial model, was then performed with time (before/after rTMS) as a within-subject factor and group (active/sham rTMS) as a between-group factor. Finally, an ROI analysis was conducted to observe if brain activity changed after iTBS over the target area (x, y, z = −50, 30, 36) during the presentation of positive and negative stimuli. Using Marsbar software (version 0.42, http://marsbar.sourceforge.net/, accessed on 23 April 2021) [53], the ROIs were defined as spheres with a diameter of 8 mm. For each subject and for each ROI, the estimated regression coefficients (beta values) were extracted before and after iTBS.

### 2.10. Sample Size Determination

The number of subjects required was determined using RStudio (pwr package). Due to the difficulty of calculating the size effect from brain imaging data when the protocol was drafted, the calculation was based on the only randomized controlled study in healthy individuals to have shown a significant reduction in BDI scores after 9 sessions of high-frequency rTMS over the left DLPFC (mean reduction: −2.33 in the active group versus −0.67 in the sham group, with an estimated standard deviation of 1.25) [21]. Assuming an alpha risk of 0.05 and a power of 0.90, we estimated the need to include 13 patients per group (bilateral test). In order to conduct statistical neuroimaging analyses and detect any significant effect of rTMS on brain activity, this number was raised to 15 patients per group.

### 2.11. Statistical Analyses

Statistical analyses were performed with SPSS software (version 26, Chicago, IL), excluding functional imaging (whole-brain) data and VBM. A repeated measures analysis of variance (ANOVA, with the time condition (before/after rTMS) as a within-subject factor and group (active/sham) as a between-subject factor) was performed to analyze variables after the equality of variances (using Levene’s test) and the normal distribution of the data (using the Kolmogorov–Smirnov test) were ascertained. When one of these tests showed significant results, non-parametric tests were undertaken (using the Mann–Whitney test). Size effects were estimated with partial eta squared (partial η^2^) when ANOVA was performed or with eta squared (η^2^) when the Mann–Whitney test was performed [54].

## 3. Results

### 3.1. Sociodemographic Characteristics

The 30 participants included (15 women, 15 men) had an average age of 25.20 years old (SD = 6.33; range = 19–45 years). At baseline, no significant difference was shown between active and placebo groups for age, gender, education level, estimated IQ and handedness (Table 2). Fourteen subjects were assigned to the active rTMS group and 16 subjects to the sham rTMS group. All participants received the intended rTMS sessions and were analyzed.

### 3.2. Clinical Measures

#### 3.2.1. Mood Scales

Clinical scales before and after iTBS are reported in Table 3. The comparison of delta scores (before minus after iTBS) did not show any significant difference between groups.

#### 3.2.2. Norris’ Visual Analogue Scales

VAS results are reported in Table S1. Repeated measures ANOVAs did not show any significant differences between groups for the 17 measured items.

#### 3.2.3. Long-term Follow-Up

Fifteen days after the end of the stimulation (*n* = 28 because of missing data for two participants in the sham group), the average HDRS score was 0.714 (SD = 1.20) in the active group and 1.286 (SD = 2.128) in the sham group. The comparison of delta scores (before minus 15 days after rTMS) did not show any significant difference between groups (U = 105.5; *p* = 0.734). Three months after the end of the stimulation (*n* = 30), the average BDI score was 0.500 (SD = 0.941) in the active group and 0.875 (SD = 2.306) in the sham group. The comparison of delta scores did not show any significant difference between active and sham groups (U = 138; *p* = 0.294).

### 3.3. Magnetic Resonance Imaging

#### 3.3.1. fMRI

fMRI data were not available for three subjects because of artifacts (one in the sham group and two in the active group). Among the 27 participants whose data were analyzed, one subject did not assess the intensity of stimuli because of a misunderstanding of the instruction.

(i) Experimental task

**Figure 3:**
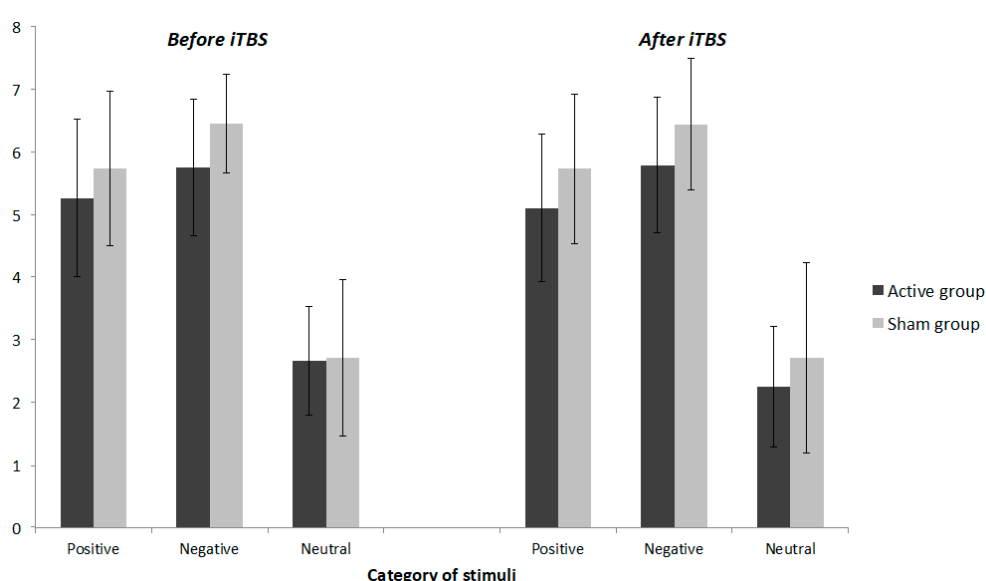
Intensity of stimuli before and after iTBS. Notes: Mean and standard deviations are reported for each group before and after iTBS. Volunteers rated each picture in terms of intensity from 1 (very low) to 9 (very high).

Figure 3 presents ratings of the intensity of the positive, negative and neutral stimuli. In the whole group, there was a significant effect of category on the intensity before (F[1:25] = 189.48; *p* < 0.001) and after (F[1:25] = 110.94; *p* < 0.001) iTBS. Before and after iTBS, negative stimuli had a higher intensity than neutral stimuli (before: t_25_ = 14.22, *p* < 0.001; after: t_25_ = 12.88, *p* < 0.001) and positive stimuli had a higher intensity than neutral stimuli (before: t_25_ = 13.77, *p* < 0.001; after: t_25_ = 10.53, *p* < 0.001), which confirmed the relevant choice of stimuli. In addition, negative stimuli had a higher intensity than positive (before: t_25_ = 2.743, *p* = 0.011; after: t_25_ = 3.405, *p* = 0.002). Regarding the effect of iTBS on intensity, no statistically significant group by time interaction effect was observed for positive stimuli (F[1:28] = 0.266; *p* = 0.610; partial η^2^ = 0.010), negative stimuli (F[1:28] = 0.029; *p* = 0.867; partial η^2^ = 0.001) and neutral stimuli (F[1:28] = 1.557; *p* = 0.223; partial η^2^ = 0.055).

(ii) Whole-brain analysis

Responses to positive stimuli (compared with neutral stimuli).

There was neither a significant group by time interaction effect nor a time effect nor a group effect.

Responses to negative stimuli (compared with neutral stimuli).

**Figure 4:**
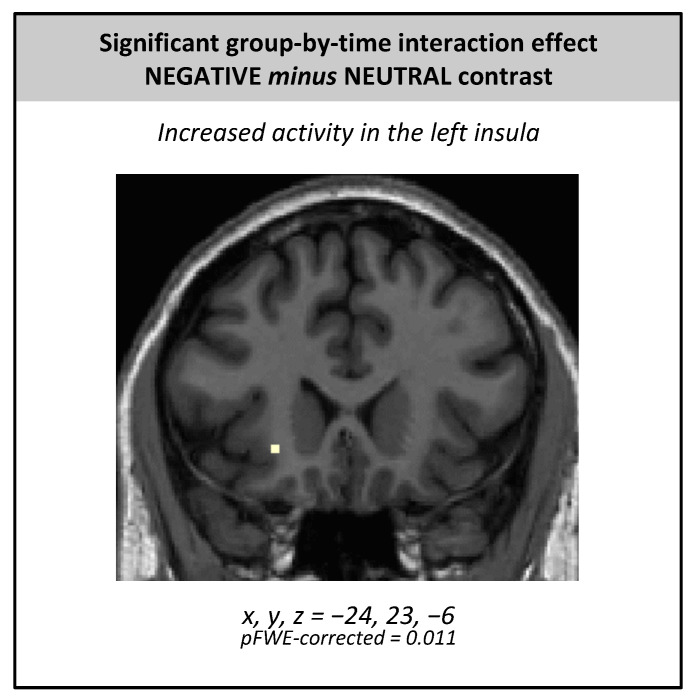
Significant group by time interaction effect in the left insula for the negative minus neutral contrast (whole-brain analysis). Notes: Coordinates (expressed in millimeters) are defined in the MNI stereotactic space (Montreal Neurological Institute, Canada).

We found a significant group by time interaction effect in the left anterior insula (x, y, z = −24, 23, −6; t = 5.63, *p*_FWE-corr_ = 0.011) with increased activity after iTBS in the active group compared with the placebo group (Figure 4). More precisely, the activity of the left anterior insula increased in 61.5% of volunteers in the active group versus 25% in the sham group (χ^2^ = 3.95; *p* = 0.047). A significant time effect was observed in the left superior temporal gyrus (x, y, z = −57, −58, 24; BA 39; t = 5.43, *p*_FWE-corr_ = 0.021) with a higher response after active/sham iTBS than before. No group effect was found.

(iii) Region of interest analysis

Neither a significant interaction effect (F[1:25] = 0.00; *p* = 0.992) nor a main effect of group (F[1:25] = 3.370; *p* = 0.078) nor a main effect of time (F[1:25] = 0.055; *p* = 0.817) was found in the rTMS target ROI (x, y, z = −50, 30, 36) for negative stimuli (compared with neutral stimuli).

Similarly, neither a significant interaction effect (F[1:25] = 0.059; *p* = 0.811) nor a main effect of group (F[1:25] = 0.571; *p* = 0.457) nor a main effect of time (F[1:25] = 0.014; *p* = 0.908) was found in the rTMS target ROI for positive stimuli (compared with neutral stimuli).

#### 3.3.2. Voxel-Based Morphometry

Analyses yielded no statistically significant group by time interaction effect nor a time effect nor a group effect at an FWE-corrected threshold (*p*_FWE-corrected_ > 0.05).

#### 3.3.3. Cortical Thickness

There was no statistically significant effect of iTBS on the cortical thickness in the left middle frontal gyrus (rostral part: U = 88.5, *p* = 0.471; caudal part: F[1:27] = 0.011, *p* = 0.916) and in the brain area that showed an activity change after iTBS, i.e., the left insula (F[1:27] = 0.594, *p* = 0.448).

### 3.4. Quality Control of the Experiment

At the end of the study, each participant answered if he/she thought that they were in the active group. Subjects thought that they were stimulated with: (i) the active coil (active group: 42.86%; sham group: 43.75%), (ii) the sham coil (active group: 35.71%; sham group: 31.25%) (iii) or they responded "no opinion" (active group: 21.43%; sham group: 25.00%). There was no statistical difference between the two groups (Fisher’s Exact test: *p* = 0.897).

### 3.5. Side Effects

Regarding side effects, Fisher’s Exact test showed no difference between the two groups. More specifically, at the end of the trial, two participants in the active group and one participant in the sham group were tired (*p* = 0.485); two participants in the active group, but no subject in the sham group, suffered from vertigo (*p* = 0.209); whereas three participants in the active group and one participant in the sham group suffered headaches (*p* = 0.345). All these adverse effects were transient.

## 4. Discussion

To our knowledge, this is the first study to explore the impact of multiple iTBS sessions on brain correlates associated with emotional processing in healthy volunteers. This study showed that 10 iTBS sessions did not induce any significant change in mood, brain activity or structure in healthy individuals, with the exception of the activation of the left anterior insula during the presentation of negative stimuli.

Regarding mood, we observed that the iTBS protocol, commonly used when treating mood disorders, had no effect (neither positive nor deleterious) in healthy volunteers. Our findings confirmed the results of two previous studies on the safety of rTMS in healthy volunteers [22,23]. However, a significant reduction in BDI scores was found after nine sessions of 25 Hz rTMS over the left DLPFC in healthy subjects [21]. An improvement in depression scores would nevertheless have been difficult to demonstrate in our study because of the very low BDI scores at baseline in both groups (less than 1), unlike in Schaller’s study, where the average scores on the 21-item BDI were around 4.

Regarding brain activity, we showed that, after receiving active iTBS, activity increased in the left anterior insula in healthy volunteers. The anterior insula is a key node in the salience network [55], in which this region could be mainly involved in the detection of relevant stimuli. In addition, the insula appears to be important in the cortical representation of internal states [56]. Thus, it could mediate the subjective experience of feelings through the representation of physiological states [57]. Over the last few years, several meta-analyses have highlighted changes in insula activity in depressive patients compared with controls [58,59,60,61,62]. Reduced insula activity was mainly demonstrated during emotional processing in depressive patients [58,59,60,62]. Hyperactivity was also, although more rarely, reported [60,61]. Given the role of the insula in the formation of the awareness of emotional states [62], this dysfunction could be associated with anhedonia in depression [58]. In addition, the insula may have a role in the modifications induced by treating mood disorders. A meta-analysis of 60 fMRI studies reported an increased activity in the left insula during positive emotions and a decreased activity in the right insula during negative emotions after patients were treated with antidepressants [63], but this effect was not shown in healthy volunteers. Two studies also showed a change in insula activity in depressed patients after high-frequency rTMS over the left prefrontal cortex [64,65]. Moreover, a clinical improvement after 4 weeks of bilateral rTMS in depressed patients was associated with decreases in functional connectivity between the prefrontal cortex and the insula [66]. The insula was also highlighted in the rare studies that included healthy participants. After a single session of 10 Hz rTMS, right insula activity decreased during the presentation of negative stimuli [16]. The apparent contradiction between this study and our findings may be explained by the difference in rTMS parameters (iTBS versus 10 Hz rTMS) or the number of rTMS sessions. The effects of several sessions of rTMS may be different from the impact of a single session, due to the probable effects of feedback and neural plasticity. Finally, a single session of iTBS applied over the left DLPFC in healthy volunteers resulted in a reduction in fronto-insular connectivity [67], suggesting that iTBS might correct an imbalance between medial and lateral frontal influence on the insula. The functional connectivity of the right anterior insula with the default-mode network also decreased 45 minutes after a single session of iTBS in healthy volunteers [34], highlighting the importance of investigating the anterior insula and, more generally, the salience network for their responsiveness to iTBS.

Finally, this study showed the safety and the good tolerability of repeated sessions of iTBS. Even with an intensity higher than that initially used by Huang (80% of rMT instead of 80% of the active motor threshold) [4], we did not observe any serious adverse effects.

### Limitations

This study has some limitations. First, the sample size was relatively small (*n* = 30), which may limit the generalization of the results. Secondly, the distribution of individuals between both groups was not even (14 versus 16 participants), due to a computer error in the randomization process. Thirdly, the absence of an effect of iTBS during the presentation of positive stimuli might be explained by their significantly lower intensity compared with negative stimuli. Consequently, the positive stimuli might not have produced a sufficiently intense emotional state. In addition, several reviews have shown that negative emotional induction is more powerful than positive induction [68,69]. A recent meta-analysis also showed that negative emotional induction presents a larger effect size for both affective valence and level of arousal in comparison with positive induction [70]. Fourth, the number of fMRI images acquired per category might not have been sufficient to demonstrate significant effects. However, this experimental paradigm has been validated in a previous study on processing emotional stimuli [71]. Finally, the main post-assessments were conducted 3 to 5 days after the last rTMS session, which is a relatively long delay. We cannot exclude a blurring of effects.

## 5. Conclusions

To conclude, this study is the first to assess the effects of 10 iTBS sessions applied over the left DLPFC (a protocol commonly used when treating mood disorders) in healthy volunteers. During the processing of negative stimuli after iTBS, activity increased in the left anterior insula, a key region that processes emotional stimuli. In future, it would be interesting to conduct a similar study on patients suffering from treatment-resistant depression, in order to identify the impact of iTBS on neural correlates of emotional processing associated with clinical improvement. Furthermore, the hypothesis of a change in connectivity between the frontal cortex and insula after several sessions of iTBS in healthy and depressed subjects should be tested.

## Figures and Tables

**Table 1:** Studies examining the effects of iTBS targeting the left DLPCF on mood and emotion processing in healthy subjects.

Study [reference]	Sample Size (M/F)	Mean Age (SD)	iTBS Protocol			Measures	Results	Side Effects
			Number of Sessions	Number of Pulses by Session	Intensity			
Pulopulos et al. (2019) [23]	35 (0/35)	23.6 (2.9) years	2	1620	110% of the resting MT	Temperament and character inventory, mood with VAS, cortisol, Trier Social Stress Test	- No effect of iTBS on mood or cortisol secretion. Higher scores on cooperativeness were associated with lower cortisol secretion, when active iTBS was administered after the social stressor.	Not reported
De Wandel et al. (2020) [31]	34 (0/34)	23.4 (3.1) years	2	1620	110% of the resting MT	Resting state fMRI, mood with VAS, cortisol, Trier Social Stress Test	- A stronger negative correlation between the left DLPFC and the caudal ACC was linked to a larger attenuation of stress-system sensitivity during active iTBS.	Not reported
De Witte et al. 2020 [32]	38 (0/38)	23.5 (3.0) years	2	1620	110% of the resting MT	Rumination, cortisol, mood with VAS, Trier Social Stress Test	- No effect of iTBS on mood. In subjects with higher levels of brooding, iTBS seemed to prevent an increase in momentary ruminative thinking and induced a reduction in cortisol secretion from a social-evaluative stressor.	Not reported
Dumitru et al. 2020 [33]	28 (17/11)	27 (6.52) years	1	600	80% of the active MT	Emotion processing tasks	- iTBS increased the recall of positive words- No effect of iTBS on negative words recall, reaction time, or accuracy in categorizing positive and negative words.	Not reported
Singh et al. (2020) [34]	26 (17/09)	28 (8) years	1	1800	80% of the active MT	Clinical assessment, mood, structural MRI, resting state fMRI	- 25 min after iTBS: reduced FC of the DMN (mainly with the rACC and dACC) - 45 min after iTBS: reduced FC of rACC and DACC; reduced FC of the DMN with right AI - Positive correlation between the FC decrease in the rACC and the harm avoidance personality trait.	No side effects

Notes: ACC: anterior cingulate cortex; AI: anterior insula; dACC: dorsal ACC; DMN: default-mode network; F: female; FC: functional connectivity; fMRI: functional magnetic resonance imaging; M: male; MRI: magnetic resonance imaging; MT: motor threshold; rACC: rostral ACC; VAS: visual analogue scale.

**Table 2:** Demographic characteristics of the sample.

	Active Group Mean (SD)	Placebo Group Mean (SD)	Statistic Value	*p*
Age (years)	24.57 (6.65)	25.75 (6.19)	t_28_ = 0.502	0.619
Gender	7 W/7 M	8 W/8 M	Χ^2^ = 0.000	1.00
Education Level (years)	15.36 (1.69)	14.50 (1.63)	t_28_ = 1.410	0.169
Estimated IQ	109.59 (5.49)	106.47 (4.64)	t_28_ = 1.654	0.110
Handedness	86.00 (9.27)	87.00 (15.04)	t_28_ = 0.209	0.836

Notes: Statistic and *p*-values correspond to the comparison between the groups. Abbreviations: SD = standard deviation; W = women; M = men.

**Table 3:** Clinical assessment: comparison of the delta scores (before minus after iTBS) between groups.

	Active Group		Placebo Group		Statistics		
	Before iTBS Mean (SD)	After iTBS Mean (SD)	Before iTBS Mean (SD)	After iTBS Mean (SD)	U	*p*	η^2^
BDI	0.57 (0.85)	0.50 (0.76)	0.25 (0.45)	0.13 (0.34)	108	0.886	0.0017
HAD	5.00 (3.40)	4.86 (2.45)	5.25 (3.53)	3.88 (2.90)	80	0.193	0.0600
HDRS	0.43 (0.65)	1.00 (1.96)	0.31 (0.70)	0.69 (1.14)	118	0.822	0.0030
MAS	0.29 (1.07)	0.36 (0.93)	0.44 (1.32)	0.94 (2.79)	131	0.448	0.0494

Notes: Mann–Whitney test compared delta scores (before rTMS minus after rTMS) between the two groups. Mann–Whitney statistical values (U), *p*-values and size effects (η^2^) are reported in the last columns. Abbreviations: SD: standard deviation; BDI: Beck Depression Inventory; HAD: Hospital Anxiety Depression Scale; HDRS: Hamilton Depression Rating Scale; MAS: Bech–Rafaelsen Mania Scale.

## Data Availability

The data presented in this study are available on request from the corresponding author. The data are not publicly available due to their containing information that could compromise the privacy of research participants.

## Supplementary Materials

The following are available online at https://www.mdpi.com/article/10.3390/jcm10112449/s1. Table S1: Norris’ Visual Analogue Scales before and after iTBS.
