# Supplementary material for: Effect of Intermittent Theta Burst Stimulation on the Neural Processing of Emotional Stimuli in Healthy Volunteers
Source: J Clin Med. 2021 Jun 1;10(11):2449. doi: 10.3390/jcm10112449 (PMC8198492; doi:10.3390/jcm10112449)
Supplement: Supplementary file 1 [file jcm-10-02449-s001.zip › jcm-1214671-supplementary.pdf]

Supplementary Table S1. Norris' Visual Analogue Scales before and after iTBS.

	Active group		Placebo group		Time effect		Group effect		Group x time interaction		
	**Before**	**After**	**Before**	**After**	**F[1:28]**	**p**	**F[1:28]**	**p**	**F[1:28]**	**p**	**Partial ηa**
Drowsiness	4.034 (1.982)	2.990 (1.969)	3.149 (2.268)	2.170 (2.231)	4.572	**0.041**	1.915	0.177	0.004	0.952	0.000
Daydreaming	3.314 (1.324)	1.780 (1.402)	2.092 (1.822)	2.090 (2.482)	3.514	0.071	0.721	0.403	3.511	0.071	0.111
Energy	6.626 (1.453)	7.602 (1.507)	7.916 (1.691)	8.664 (1.662)	6.708	**0.015**	6.093	**0.020**	0.117	0.735	0.004
Clarity of mind	8.020 (1.308)	8.719 (1.157)	8.630 (1.347)	9.122 (1.123)	14.587	**0.001**	1.416	0.244	0.441	0.512	0.016
Clumsiness	3.180 (1.951)	2.010 (1.157)	2.110 (1.942)	1.010 (1.109)	14.89	**0.001**	3.478	0.073	0.020	0.889	0.001
Vivacity	7.368 (1.174)	8.336 (1.280)	8.278 (1.628)	8.754 (1.334)	12.384	**0.002**	2.095	0.159	1.437	0.241	0.049
Weakness	2.980 (1.308)	2.180 (1.684)	2.590 (2.488)	1.160 (1.379)	6.920	**0.014**	1.973	0.171	0.552	0.464	0.019
Boredom	2.280 (1.565)	1.332 (1.329)	1.470 (1.615)	1.539 (1.832)	3.963	0.056	0.306	0.585	5.217	**0.030***	0.157
Competency	8.097 (1.402)	8.712 (0.989)	8.940 (1.151)	9.194 (1.002)	8.998	**0.006**	2.851	0.102	1.542	0.225	0.052
Sadness	2.530 (1.913)	1.860 (1.081)	1.460 (1.320)	1.470 (1.633)	1.504	0.230	2.259	0.144	1.545	0.224	0.052
Peace of mind	7.625 (1.781)	8.662 (0.791)	8.791 (1.383)	9.198 (1.006)	6.853	**0.014**	4.933	**0.035**	1.303	0.263	0.044
Dissatisfaction	2.490 (1.420)	1.650 (1.150)	2.080 (2.386)	0.890 (0.992)	7.644	**0.010**	1.648	0.210	0.227	0.638	0.008
Sociability	7.864 (1.604)	8.382 (1.115)	8.973 (1.018)	9.416 (0.799)	6.136	**0.020**	8.139	**0.008**	0.037	0.849	0.001
Restlessness	3.852 (1.588)	2.470 (1.878)	2.728 (1.937)	2.000 (2.171)	6.703	**0.015**	1.958	0.173	0.641	0.430	0.022
Relaxation	6.950 (1.668)	8.260 (1.200)	8.330 (1.189)	9.090 (1.093)	16.804	**0.001**	7.647	**0.010**	1.172	0.288	0.040
Quality of life	7.720 (1.703)	8.390 (1.350)	8.830 (1.564)	8.810 (1.668)	3.255	0.082	1.940	0.175	3.645	0.067	0.115
Coping	8.110 (1.406)	8.590 (1.109)	8.890 (1.490)	8.920 (1.697)	2.976	0.096	1.188	0.285	2.337	0.138	0.077

Notes: Mean (SD) are reported for each group before and after iTBS. Statistic and p values are reported for different factors of the repeated measures analysis of variance. Size effects (partial η2) are specified for group x time interaction effect. Values in bold indicate $p \leq 0.05$. * This result was no longer significant after Bonferroni correction for multiple comparisons.
